# Neurovascular Coupling by Functional Near Infra-Red Spectroscopy and Sport-Related Concussion in Retired Rugby Players: The UK Rugby Health Project

**DOI:** 10.3389/fnhum.2020.00042

**Published:** 2020-02-13

**Authors:** Anick Sharma, Karen Hind, Patria Hume, Jyotpal Singh, J. Patrick Neary

**Affiliations:** ^1^Independent Researcher, Durham, United Kingdom; ^2^Department of Sport and Exercise Sciences, Durham University, Durham, United Kingdom; ^3^Sports Performance Research Institute New Zealand (SPRINZ), Faculty of Health and Environmental Science, Auckland University of Technology, Auckland, New Zealand; ^4^Faculty of Kinesiology and Health Studies, University of Regina, Regina, SK, Canada

**Keywords:** brain, concussion, contact sport, neuroimaging, retired athletes

## Abstract

**Aim**: This study investigated cerebral hemodynamic responses to a neurovascular coupling (NVC) test in retired contact athletes with a history of repeated mild traumatic brain injury (mTBI) and in controls with no history of mTBI.

**Methods**: Twenty-one retired rugby players (47.7 ± 12.9 year old; age at retirement: 38.5 ± 8.9 year; number of years playing rugby: 12.7 ± 3.7 year) with a history of three or more diagnosed concussions (8.9 ± 7.9 concussions per player) and 23 controls with no history of mTBI (46.5 ± 12.8 year old) performed a NVC test to detect task-orientated cerebral hemodynamic changes using functional near-infrared spectroscopy (fNIRS).

**Results**: The NVC showed a statistically significant reduction in the cerebral hemodynamic response in comparison to the control group which had a greater relative increase of oxyhemoglobin (O_2_Hb). There were reductions in left middle frontal gyrus (MFG) O_2_Hb (−0.015 ± 0.258 μM) and relative increases in deoxyhemoglobin (HHb; −0.004 ± 0.159 μM) in the same region for the mTBI group in comparison to the control group (−0.160 ± 0.311 μM; −0.121 ± 0.076 μM for O_2_Hb and HHb, respectively). The mTBI group induced a greater rate of oxygen extraction compared to the control group.

**Conclusion**: This was the first study to examine cerebral hemodynamic changes in retired rugby players in response to a NVC test, and we found reduced cerebral hemodynamic responses in participants with a history of mTBI compared to controls. These results suggest altered cerebral metabolic demands in participants with a history of multiple head injuries. Further research is needed to ascertain an understanding of the changes in hemodynamics from playing into retirement.

## Introduction

Concussion or mild traumatic brain injury (mTBI) arises from a sudden movement of the brain within the cranium, with rapid angular or linear acceleration, deceleration or rotational force (Jordan, 2013). The clinical presentation of concussion can include loss of consciousness, altered mental state, nausea, headaches, vertigo, and amnesia. Participation in contact sports increases the risk of concussion (La Fountaine et al., 2016), and sports-related concussion is one of the most common injuries reported in rugby union players in the UK (Hume et al., 2017; Sport England, 2017). Over the course of one competitive season, rugby players have been reported to encounter 600–800 body impacts and around 50 to over 100 direct head impacts (King et al., 2017). Consequently, this has resulted in urgent calls for research to improve the understanding of risks, methods for diagnosis, and long-term brain consequences for those athletes affected (Lipnick et al., 2018). Understanding the long-term effects of sports-related concussion is a current challenge. It has been suggested that repetitive concussive injuries increase the risk of neurodegenerative problems in later life ranging from mild cognitive deficits to neurological disease such as Alzheimer’s (McKee et al., 2009; Johnson et al., 2010; Omalu et al., 2011).

The multifaceted pathophysiology of concussion varies across the acute to longer-term phases and can involve endothelial cell impairment of the blood-brain barrier, mitochondrial dysfunction which may lead to the disruption of cerebral autoregulation and cerebral blood flow velocity (Len et al., 2013; La Fountaine et al., 2016; Yang et al., 2017; Neary et al., 2019). Cerebral vasoconstriction leads to reduced arterial oxygen and consequently, there are blood pressure changes to preserve brain oxygen (Curtelin et al., 2017). Resultantly, cognitive impairment and physical capability can become diminished from this process.

Under normal healthy conditions, neural stimulation can induce an increase in oxyhemoglobin (O_2_Hb) and a decrease in deoxyhemoglobin (HHb). Taken together, these changes imply increases in local arteriolar vasodilatation, leading to increases in local cerebral blood flow and blood volume, and metabolic changes at the cellular level (Ferrari and Quaresima, 2012). This blood volume, when measured using functional near-infrared spectroscopy (fNIRS) can be indicated by the sum of O_2_Hb and HHb, known as total hemoglobin (tHb = O_2_Hb + HHb). To understand the extraction of oxygen during neural stimulation, the hemoglobin difference (HbDiff = O_2_Hb – HHb) can be calculated and can reflect metabolic cellular changes. Repeated head impacts can hinder the relationship between these neurometabolic parameters (Bailey et al., 2013). An increase in regional brain activity implies an increase in metabolism, thereby requiring greater blood flow to the area (Tan et al., 2014), hence providing evidence for coupling between cerebral blood flow and metabolism. This complex interaction of neurons, glia, and vascular cells responsible for delivery of blood to the metabolically targeted area is termed neurovascular coupling (NVC; Girouard and Iadecola, 2006). Repeated head exposure can potentially uncouple this process (Richards et al., 2001), perhaps endothelial dysfunction can lead to this neurovascular dysfunction (Tan et al., 2014).

fNIRS is a non-invasive, optical, neurophysiological imaging technique that can be used to detect relative changes in cerebral oxygenation. Thus, it has the potential to be used to examine several physiological mechanisms including NVC (Ellis et al., 2016; Epps and Allen, 2017). Few studies have used fNIRS for the assessment and monitoring of post-concussive injury (Urban et al., 2015; Bishop and Neary, 2018; Hocke et al., 2018). Relative changes were observed over time with O_2_Hb decreasing after the initial concussion injury and HHb increasing until day 14 post-injury. Forcione et al. (2018) reported similar changes that were validated with fMRI, also within a current group of playing athletes. The results suggest that fNIRS is a valid tool when assessing concussion in athletes undergoing return-to-play protocols.

To date, it is unclear what the long-term effects are on the neurophysiology in retired rugby players with a career of repeated head injuries. Therefore, the purpose of this study was to investigate NVC responses in a cohort of former rugby players who had a history of sport-related concussion, compared with a control group who had no history of concussion.

## Methodology

### Study Design and Research Ethics

This study was conducted as part of the multi-disciplinary UK Rugby Health Project which was designed to investigate the health of retired rugby players. The current study focused on the NVC cerebral hemodynamic responses and history of sports-related concussion. The study was reviewed and approved by the University Research Ethics Committee and all participants provided their signed informed consent prior to testing. Ethical approval for the research was granted by the Carnegie Faculty Research Ethics Committee, and the research was carried out in accordance with the Declaration of Helsinki (2013).

### Participants

Participants were recruited from the UK Rugby Health Project, launched in September 2016 with the primary objective of improving the understanding of long-term health in retired rugby players. The full demographics of all the participants can be seen in Table 1. The former rugby players are comprised of elite and community-level athletes who had a history of sports-related concussions. Inclusion in this sub-study was based on their responses in the General Health Questionnaire (GHQ) and their availability to take part in clinical testing. Forty-four participants were recruited from the United Kingdom, with 21 participants in the mTBI group being former male rugby players, and 23 non-contact sport participants in the control group (former cricket players, runners, or no competitive sport). Retired rugby participants were drawn from both rugby union and rugby league codes, and had experienced at least three confirmed sports-related concussions. Participants were recruited from past player associations, word of mouth and social media. The control group participants may have played the sport but must not have competed in any contact sport since the age of 16 years and reported no history of concussion. All participants completed a GHQ which included a section on injury and concussion.

**Table 1:** Descriptive statistics of mTBI and control groups (*denotes significant difference between mTBI and control, *p* < 0.05).

Variable	mTBI (frequency or mean ± SD)	Control (frequency or mean ± SD)
Participants	21	23
Age (years)	47.7 ± 12.9	46.5 ± 12.8
Height (cm)	180.1 ± 8.3	177.9 ± 7.1
Current body mass (kg)	101.1 ± 17.3*	77.4 ± 18.6
Main sport	Rugby Union: 16 Rugby League: 3 Not recorded: 2	Cricket: 5 Football: 3 Mountaineering: 1 Gym: 6 Running: 8
Highest sport level	Amateur: 11 Professional: 8 Not recorded: 2	Amateur: 23 Professional: 0
Years played sport	12.7 ± 3.8	15.7 ± 3.1
Playing body mass (kg)	95 ± 15.1	81.2 ± 11.1
Smoking status	Never smoked: 14 Ex-smoker: 3 Occasional smoker: 1 Regular smoker: 1 Not recorded: 2	Never smoked: 12 Ex-smoker: 7 Occasional smoker: 4
Alcohol status:	Non-drinker: 2 Monthly or less: 3 Two to four times per month: 4 Two to three times per week: 5 Four or more times per week: 4 Not recorded: 2	Non-drinker: 6 Monthly or less: 10 Two to four times per month: 4 Two to three times per week: 3

### Procedures

All testing took place in the same temperature-controlled (22.5°C) room, with no distractions. Participants were hydrated and had not exercised or consumed caffeine and alcohol within the preceding 12 and 24 h, respectively. Standing height and body mass (stadiometer and scales SECA, Birmingham, UK) were measured and body mass index was calculated.

#### fNIRS Device and Protocol

The fNIRS cranial headpiece was placed 1 cm above the eyebrow on the supraorbital ridge to avoid the sinuses. The 8-channel system (OctaMon, Artinis Medical, Netherlands) monitored changes in O_2_Hb and HHb in the pre-frontal cortex, specifically covering the dorsolateral (DLPFC) and orbitofrontal (OFC) cortices in both hemispheres (Vergotte et al., 2018).

Our study performed a modified Neary Protocol (Neary et al., 2019) to assess NVC using the “Where’s Wally (Waldo)” paradigm. Previous research has confirmed this test is a valid measure of NVC (Smirl et al., 2016). To start the Neary Protocol, the participant sat quietly in a chair with both feet flat on the floor, for 5 min, breathing normally with their eyes open and focussed looking straight forward. The researchers did not communicate with the participant during this baseline data collection period. The “Where’s Wally” test (Smirl et al., 2016; Neary et al., 2019) was then implemented on a plasma screen (42" size), with the participant sitting 180 cm away. Each of the five cycles (5 min in total) consisted of 20 s of eyes closed followed by 40 s of eyes open where the participant searched the screen for “Wally.” If the participant found “Wally” within the 40 s then the slide was immediately advanced to the next slide where they had to try to find “Wally” again but in a different backdrop.

#### fNIRS Outcome Measures

**Figure 1:**
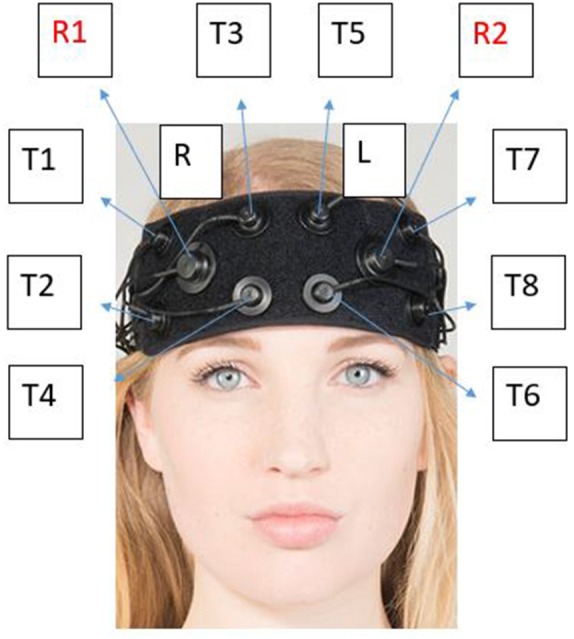
Corresponding “T regions” to the OctaMon channels. Each “T region” represents the channel in which light detected using near-infrared spectroscopy (NIRS) between the left and right side. The channels emit photons from the light emitter which is then absorbed into the skull and received back into receiver 1 (R1) or receiver 2 (R2). The known penetration depth can then be used to calculate relative changes in neurometabolic parameters. Neuronavigation using the 10-20 system is used as per the manufacturer optode arrangement. The optodes (light emitter) are separated by 3 cm from each other and 3.5 cm from the receiver whilst penetrating 1.5 cm into the skull. This photo has been modified with express permission from Artinis.

The neurometabolic parameters (O_2_Hb, HHb, tHb and HbDiff) were measured *via* the OctaMon fNIRS (OctaMon, Artinis Medical System, Netherlands) *via* the absorption of attenuating light. All parameters were measured using 765 and 855 nm penetrating wavelengths with an emitter to optode distance of 3.5 cm resulting in a penetrated tissue of approximately 1.5–1.75 cm. The 8-channel system recorded the hemoglobin signals at 10 Hz (Wightman et al., 2015). Different channels, or “T regions” (Figure 1) correspond to different regions of the OctaMon placement on the forehead. Relative concentration changes (μM) were calculated with the manufacturer’s software using a modified Beer-Lambert law (Obrig and Villringer, 2003) which examined the attenuation of light depending on the absorbance of the material. The Artinis software v.3.0.95 (Oxysoft Artinis Medical, Netherlands) calculated relative changes between the two main chromophores from the optical density signals depending on the different wavelengths for O_2_Hb and HHb and adjusted the signals according to the differential pathlength factor (DPF) calculated by age (Duncan et al., 1996).

#### Data Analyses

Data were imported at 10 Hz into Microsoft Windows Excel. Analysis began by graphing each participant against time, pairing up two channels at a time. One individual (mTBI) had to be excluded from any further analysis due to the signal cutting mid-way and ending up with too much noise to differentiate any changes. Outputs were then reviewed to remove any artefacts and noise to leave a clean dataset. Once this was completed for each participant in the subsequent group (control vs. mTBI), all data were compiled together on a custom-made master template which averaged each neurometabolic parameter for each channel. For example: channel 1 was averaged for O_2_Hb, HHb, tHb and HbDiff for mTBI individuals, then for controls, then channel 2 was averaged for O_2_Hb, HHb, tHb and HbDiff, until all eight channels had been averaged and two datasets remained; control and mTBI. An additional template was created for “control vs. mTBI” which displayed a full graphical representation per channel per-protocol segment. Further outputs were then created from templates that were relative to change from baseline (5-min rest).

Statistical analysis was performed using IBM SPSS Statistics v.22.0 software (SPSS INC., Chicago, IL, USA). The averages of the relative change for O_2_Hb, HHb, tHb, and HbDiff were used. Shapiro-Wilk tests were conducted to identify distribution and a one-way ANOVA was performed to see any differences between channels within groups with the homogeneity of variance (Lavene test) also conducted. Bonferroni *post hoc* tests were performed to investigate pairwise comparisons. A mixed ANOVA was then performed to see if there were any differences between groups. Sphericity was checked, and the Greenhouse-Geisser correction was used if sphericity was violated. Pairwise comparisons were conducted on the mixed ANOVA between the channel and comparative neurometabolic parameters between groups. Statistical significance was set at *p* < 0.05.

## Results

### Descriptive Data

Table 1 presents the demographics for the mTBI and control groups alongside the type and level of rugby played. Non-contact sports played for the controls are also displayed. The only demographic difference between the groups was in playing body mass. The significant difference between groups in playing body mass was likely due to the physiological demands of rugby in comparison to non-contact sports.

Table 2 shows the mTBI group descriptive results further with positional play in both rugby union and league. Concussion history is also presented during time spent playing the respective rugby code, symptomology and return to play.

**Table 2:** mTBI descriptives.

Variable		Frequency or years ± SD
Number of retired players with a history of concussion		21
Number of players retired due to injury		11
Mean retirement age		38.5 ± 8.9
Rugby union positions:		
	Front row	4
	Second row	2
	Back row	3
	Halfbacks	2
	Centres	3
	Back three	4
Rugby league positions:	Scrum-half	1
	Second row	1
	Prop	1
Number of times previously reported concussions:		
	3–5	12
	6–9	3
	10–14	2
	15–20	3
	20+	1
Number of concussions sustained outside of rugby:		
	None	16
	One	5
Number of ex-players currently affected by concussion symptoms		6
Number of times reportedly knocked out whilst playing:		
	Never	6
	Once	4
	Twice	2
	Three	3
	Four	2
	Six	2
	Seven	2
Return to play/competition on the same day of an injury/concussion on separate occasions:		
	Never	3
	Two	6
	Three	2
	Four	1
	Five	1
	Six	1
	Seven	1
	Ten	6
Number of presently persisting symptoms post retirement:		
	Headaches and migraines	4
	Sleepiness	7
	Irritability	7
	Depression	6
	Anxiety	6
	Memory loss	6

### Neurovascular Coupling

A between channel one-way ANOVA showed significant changes (*F*_7_ value; *p* value) for mTBI for O_2_Hb (*F*_7_ = 4,700, *p* < 0.01), HHb (3,175; < 0.01), tHb (3,175; < 0.01) and HbDiff (15,166; <0.01). Greenhouse-Geisser showed significant changes between O_2_Hb, HHb. tHb and HbDiff between the channels and groups: *F*_(7,11.5)_ = 1,865,735, *p* < 0.05. Pairwise comparisons indicated that the control O_2_Hb had a higher relative change in channels 1–4, 7 and 8. The same group also had a greater relative change of HHb and HbDiff across all channels in comparison to the mTBI group (*p* < 0.01).

**Figure 2:**
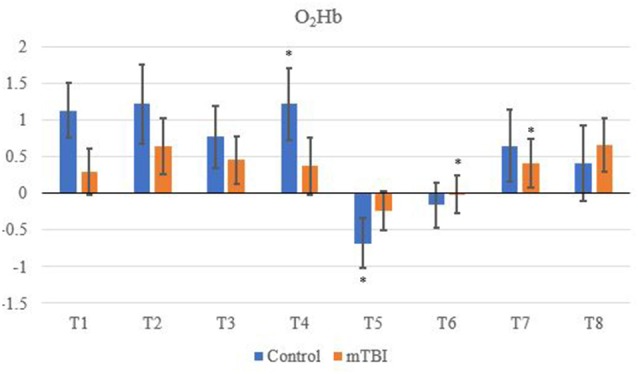
Mean O_2_Hb neurovascular coupling (NVC) relative change (μM) from rest for all eight channels (represented by T1–T8) for mild traumatic brain injury (mTBI) and control participants. Relative change refers to the occurred differential from baseline for the measured neurometabolic parameter (*denotes significant difference between the mTBI and control groups for the respective channel, *p* < 0.01).

**Figure 3:**
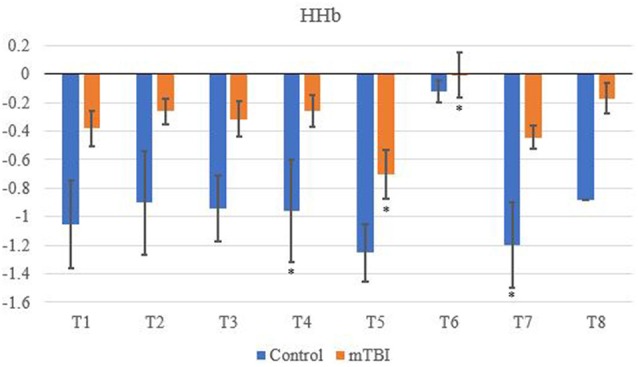
Mean HHb NVC relative change (μM) from rest for all eight channels (represented by T1–T8) for mTBI and control participants. Relative change refers to the occurred differential from baseline for the measured neurometabolic parameter (*denotes significant difference between the mTBI and control groups for the respective channel, *p* < 0.01).

**Figure 4:**
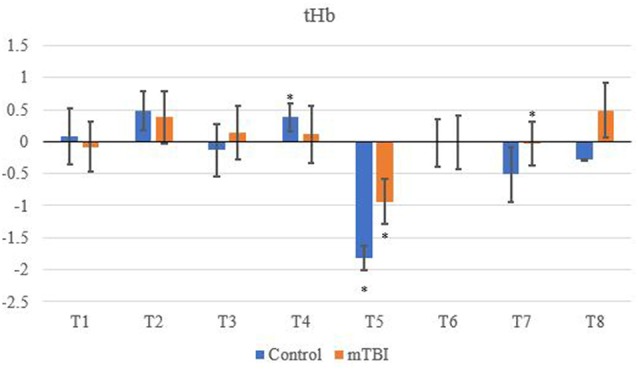
Mean tHb NVC relative change (μM) from rest for all eight channels (represented by T1–T8) for mTBI and control participants. Relative change refers to the occurred differential from baseline for the measured neurometabolic parameter (*denotes significant difference between the mTBI and control groups for the respective channel, *p* < 0.01).

**Figure 5:**
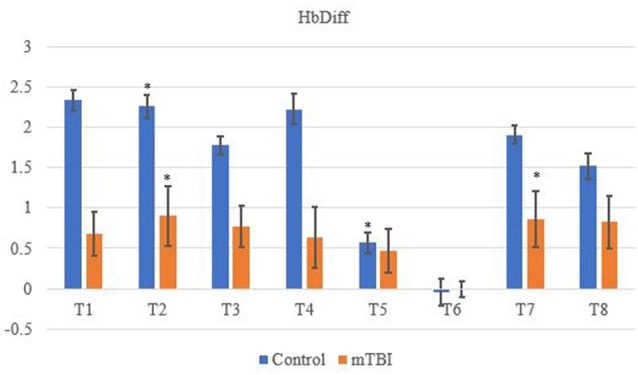
Mean HbDiff NVC relative change (μM) from rest for all eight channels (represented by T1–T8) for mTBI and control participants. Relative change refers to the occurred differential from baseline for the measured neurometabolic parameter (*denotes significant difference between the mTBI and control groups for the respective channel, *p* < 0.01).

Significant changes were shown for the mTBI group between the neurometabolic parameters within group: O_2_Hb (*F*_7_ = 280,008, *p* < 0.01), HHb (529,594; < 0.01), tHb = (388,308; < 0.01) and HbDiff (145,144; < 0.01). *Post hoc* analysis revealed O_2_Hb channel 7 was significantly the highest and channel 6 was significantly the lowest (Figure 2). HHb was the lowest in channel 5 and highest on channel 6 (Figure 3). tHb was significantly the lowest in channel 5, and the highest in channel 7 (Figure 4). HbDiff had a significant relative change from rest in channel 2 for the mTBI group, and channel 5 for the control group (Figure 5, *p* < 0.01).

For the control group, the *post hoc* analysis revealed O_2_Hb and HHb were the highest in channel 4 (Figures 2 and 3, respectively), whilst O_2_Hb and tHb were the lowest in channel 5 (Figures 2 and 4, respectively).

## Discussion

This is the first study to the authors’ knowledge which explored neurometabolic changes and the NVC mechanism in multiple areas of the prefrontal cortex of retired rugby players with a history of concussion in comparison to retired athletes with no prior history. The most significant results of this study showed that channel 6 had the greatest reduced relative cerebral hemodynamic response (decrease O_2_Hb and increased HHb) out of all eight regions for the mTBI group. This reflected changes in the middle frontal gyrus (MFG).

### Between-Group Differences in fNIRS Outcome Measures

Control tHb elicited a greater volume of change in comparison to mTBI (Figure 4). If O_2_Hb is not changing, then HHb must be matched for an increase in tHb for comparison to baseline. This was apparent as HHb values (Figure 3) decreased from the rest. This was further evidenced as mTBI O_2_Hb had the lowest relative change in the OFC, specifically the left MFG (channel 6, Figure 2), whereas HHb was the highest for the same channel (region). This suggests that the mTBI group had significantly less O_2_Hb and significantly more HHb in the left MFG. The mTBI group had their left caudal PFC (channel 7) as the strongest O_2_Hb signal. This coincides with literature (Oudegeest-Sander et al., 2014; Hirose et al., 2016) as they both measured hemodynamics and reported tHb will be greater at baseline for the control group as mTBI individuals will have overcompensation of HHb from a lack of O_2_Hb, which may be indicative of conditions such as ischemia. From a practical perspective, this is telling of impacts to the prefrontal cortex as channel 6 is over the left MFG. Wu et al. (2018) demonstrated increased activation of the left MFG within mTBI individuals. The results of this study support this as increased HHb activation has been recorded within the mPFC. Consequently, regions of the brain that have been recorded to be impaired are primarily responsible for cognition and behaviour (Johnson et al., 2010). This may lead to the possibility of changes in personality from reduced cerebrovascular hemodynamics. However, this is presumptive as no histories of areas of impacts were recorded from the mTBI group, albeit, it provides insight into susceptible areas of impact.

The decrease in O_2_Hb and an increase in HHb within the left MFG is supported by Bailey et al. (2013). Similar increases of HHb were demonstrated in current professional boxers with reported mTBIs, as was the case in the present study. However, changes through an NVC task supports evidence that HHb differences are regulated further by neurogenic mechanisms (Tachtsidis and Scholkmann, 2016; Cheng et al., 2017). On the contrary, the control group did not produce a similar relationship between O_2_Hb and HHb.

tHb had the lowest signal in channel 5 (dorsolateral PFC) for both groups, meaning tHb (Figure 4) is matched for the NVC response, but differences in O_2_Hb and HHb hemodynamics do occur. This is supported in the literature (Hirose et al., 2016; Curtelin et al., 2017; Hocke et al., 2018) as tHb is equated within controls which are a compensatory mechanism from the change between O_2_Hband HHb resulting in a reduction of variability for mTBI individuals. Similar findings have been observed in the standard deviation of O_2_Hb using NIRS in a group of mTBI athletes following acute concussion (Bishop and Neary, 2018).

Changes in O_2_Hb and HHb are possibly attributed to the blood-brain barrier as changes in permeability may cause increases in intracranial pressure and cerebral perfusion pressure (CPP). The amount of arterial O_2_Hb decreases from the higher tensions of the cerebrovascular wall and causes an increase in HHb (Curtelin et al., 2017) through diminished cerebral autoregulation. Further explanations of BOLD fMRI signalling can be used to explain this as HHb typically decreases with an association from increased CBF. O_2_Hb delivery exceeds the amount consumed through neuronal activation which propagates proportional changes between O_2_Hb and HHb (Ellis et al., 2016; Orhan et al., 2016). However, Figures 2, 3 show that O_2_Hb decreases and HHb increase simultaneously in the left MFG during the NVC task in the mTBI group, which would be associated with neuronal activation. The mTBI group showed a greater HbDiff decrease (Figure 5) within the same area which increased the oxygen extraction. This can be attributed to the neuronal activation (increased HHb). Two mechanistic outcomes can come possibly explain this; either neuronal activation is impaired, or the observed effect was once again within channel 6, the left MFG, which relates to impact at the front of the head. Resultantly, regional links can be established, and the consequential neurophysiological changes can be seen to impact “performance.” Recent research has shown that cortical inhibition can occur in post-concussion syndrome (Pearce et al., 2019) and may have acute (as well as longer-term) impacts on the brain (Di Virgilio et al., 2016) which would support our research. Thus, questions are raised regarding pressure changes in the microvasculature within the set population. If the protocol evokes such deleterious changes to hemodynamics in a relatively middle-aged sample, further considerations need to be sought for aging populations who have had a lifetime of rugby exposure, or in athletes playing other contact sports.

Channel 4 (medial PFC) was the highest for both O_2_Hb and HHb for the control group (Figures 2, 3). The lowest signals for O_2_Hb and HHb were the dorsolateral LPFC and caudal PFC. This can be attributed to minimal differences in both the outcome and relative location of the channel detector. Thus, in support of Liboni et al. (2009), O_2_Hb changes are marginal within the NVC task for a control group as functional microvasculature is free from pressures that are paramount in mTBI cases, consequential of a lack of impaired hemodynamics (Jindal et al., 2015). This also explains why tHb did not have a large magnitude change and stayed relatively consistent, especially within the NVC task, as it did not have to be matched from increasing amounts of HHb relative to O_2_Hb intra-channel. However, this was from examining task-orientated changes, where the general cerebral hemodynamic perturbations could induce responses for both groups. Ferreri et al. (2014) suggested that the greatest cerebral hemodynamic changes happen from task orientated protocols, and thus we used the “Where’s Wally” paradigm for this function. The literature further bolsters that it can be more than generic changes as Akın and Bilensoy (2006) reported HHb increases within protocols that induce metabolic perturbations. Strangman et al. (2017) used astronauts and a head tilt to evoke pressure changes, whereas Akın and Bilensoy (2006) looked at changes within migraine patients whilst looking at NVC components.

The magnitude of change within O_2_Hb and tHb is also confirmed in literature; the change in magnitude is less in mTBI than a control group (Kontos et al., 2014; Helmich et al., 2016; Hocke et al., 2018). Additionally, Hocke et al. (2018) reported the greatest changes in the left DLPFC when investigating functional connectivity in mTBI participants. This supports the current findings as the largest changes have been observed in the left DLPFC from channel 6 onwards.

### Difference Between Neurovascular Coupling Outcomes Within Groups

Increases in O_2_Hb were apparent within the caudal PFC, ventrolateral PFC, dorsolateral DLPFC and medial PFC which coincides with increases in tHb for those channels. Alongside decreases in HHb throughout the NVC protocol, Phillips et al. (2015) also showed similar neurometabolic differences. Results were indicative of activation of the occipital lobe which is triggered by repetitive visual stimuli as was the case in this study. While full mechanisms are unknown (Schytz et al., 2019), such changes in cortical vasculature can be attributed to neuronal activation for hyperaemic responses through the Circle of Willis. As the two main arteries; internal carotid and vertebral are responsible for maintaining CPP, if one is disrupted (as may be the case in an mTBI) then perivascular neurons from both sympathetic and parasympathetic systems would be needed to maintain pressure (Purkayastha et al., 2019). Furthermore, mTBIs can cause damage to large arteries and impede blood flow, which may explain the difference between control and mTBI. Whilst this is more indicative of acute mTBI, physiological degenerations within the brain can occur later (Kaushal et al., 2019). The trauma can lead to oxidative stress which may cause changes in CPP and a resultant negative connotation with NVC. This may be due to the inversed neuronal activation which causes a hyperaemic response.

The changes that occurred during this protocol may also be attributed to intracellular and extracellular hemodynamics. Neuronal activated tasks may evoke cerebral hemodynamic changes (false positive) or change neuronally induced neurometabolic changes (false negative). If this was the case, equipment needed to be scrutinised and validated as wavelength combinations can create “crossing” between chromophores; positive changes in one may produce a negative elsewhere (Tachtsidis and Scholkmann, 2016). While this point was considered, careful experimental design alongside unnecessary systemic activation and stress minimises this risk. fNIRS has been validated against gold standards (Alderliesten et al., 2014; Yang et al., 2017) despite the sensitivity to extracerebral compartments. However, this was not necessarily a negative point as non-neuronal changes occur and can be indicative of links between NVC and cardiometabolic factors within the extracerebral space. Although CBF is further regulated by O_2_Hb for the ANS, HHb is further contaminated within the extracerebral compartment. Increases in cerebral blood flow can be associated with increases in neural activity, and the large increase in cerebral blood flow as shown in “Where’s Wally” as compared to reading or observing coloured dots. This implies that “Where’s Wally” can be used to assess NVC (Smirl et al., 2016). Using fNIRS in this study provided more hemodynamic detail to the cerebral blood flow changes which are associated with NVC. It has been suggested that changes in cerebrovascular function following concussion can be the underlying physiological reason behind the associated concussion symptoms (Tan et al., 2014), and there is very limited human data available to associate neurophysiological changes over time due to prior concussions.

### Limitations of the Study

There were several limitations to the study that we acknowledge. First, it is important to note that fNIRS provides a relative change from the delta values, and can be very different from individual to individual. These subjective differences could potentially be nullified because of this as there is more variability to the data. Additionally, some individuals in the current study were aged 50 year or older, and the DPF calculated does not consider any age after 50 years. Therefore, the light scattering medium could potentially be underestimated, so results could be of a greater magnitude. However, participants in each group were of approximately the same age, and this means this age issue is not likely a concern for our data.

As previously mentioned, the sample was male participants only as there are more retired male players than females. There is a need for the study to be repeated in female retired players to investigate if similar findings exist or if there are sex-specific differences. Additionally, the study used convenience sampling, with selection bias issues acknowledged. The impact of this was lessened as participant selection was completed through the UK Rugby Health Project, blinding any bias once eligibility had been determined. The data on concussion were based on self-report, and therefore there may have been inaccuracies from individual participant reported numbers of concussions. For this reason, no analyses were conducted between specific number of concussions and neurovascular outcomes.

No simultaneous blood pressure was obtained in the study and no task-based cognition test was administered. This would have enabled the study to evaluate further pressure changes that have been associated with cognitive NVC. However, NVC was stimulated by a sufficient protocol segment meeting the previously mentioned criteria.

## Conclusions

In conclusion, retired players with a history of mTBIs display altered cerebral hemodynamic responses in comparison to a control group of similar-aged retired athletes without a history of concussion. O_2_Hb was less in the caudal PFC, DLPFC and left MFG in mTBI individuals, while they also had a higher amount of HHb within the left MFG through the NVC protocol. The left MFG showed significant differences in hemodynamics (higher amounts of HHb and lower amounts of O_2_Hb) compared to controls suggesting an altered NVC cerebral autoregulatory mechanism which may be indicative of susceptibility of impact area when an mTBI is experienced.

Area-wide differences between protocol segments generally showed controls elicited higher rates of O_2_Hb, and mTBI individuals showed greater HHb. tHb decreased throughout indicating changes of vasoreactivity and may explain why the control group had a greater change of O_2_Hb, as they have not experienced cerebrovascular dysregulation.

### Future Directions and Implications

There is a need for longitudinal research that monitors neurovascular function from the middle of a player’s career and through retirement. Further studies may look to investigate impact areas of the head and long-term monitoring of the left MFG post-retirement. As the field of cerebral hemodynamic research is an emerging one, a longitudinal study would give a comprehensive understanding to the neurovascular involvement and longer-term outcomes of concussion. Such research could take on board the considerations outlined; with continuous blood pressure, cognition changes with task-orientated protocols and advances in current technological software to further monitor changes at 3-year intervals post-retirement. By such a time, the numbers of retired female players will have increased, leading the way for further research into this area. There has been an increase of anecdotal reports of other sports such as football (soccer), with head injuries becoming more widespread, with very recent developments in the elite form. This can lead to more sports conducting research to look at both short term and long-term effects, using a novel approach such as ultra-sound tagged NIRS.

## Data Availability Statement

The raw data supporting the conclusions of this article will be made available by the authors, without undue reservation, to any qualified researcher.

## Ethics Statement

The studies involving human participants were reviewed and approved by the Research Ethics Committee of Leeds Beckett University. All patients/participants provided their written informed consent to participate in the study.

## Author Contributions

AS, KH, and JN contributed to the study concept, design and data collection. KH contributed as postgraduate supervisor for AS, and overall lead for the UK Rugby Health Project. AS and JS contributed to the data analysis. All authors contributed to the drafting of the article.

## Conflict of Interest

The authors declare that the research was conducted in the absence of any commercial or financial relationships that could be construed as a potential conflict of interest.
